# A new online detection method of tobacco impurities for tobacco robot

**DOI:** 10.3389/fnbot.2024.1422960

**Published:** 2024-06-07

**Authors:** Lei Zhang, Dailin Li, Dayong Xu, Erqiang Zhang, Zhenyu Liu, Jiakang Li, Jinsong Du, Shanlian Li

**Affiliations:** ^1^Key Laboratory of Tobacco Processing Zhengzhou Tobacco Research Institute of CNTC, Zhengzhou, China; ^2^College of Electrical and Information Engineering Zhengzhou University of Light Industry, Zhengzhou, China; ^3^Technology Center, China Tobacco Shanxi Industrial Co., Ltd., Xian, China

**Keywords:** tobacco industry, tobacco robot, real-time processing, automated inspection, tobacco impurity detection

## Abstract

In the tobacco industry, impurity detection is an important prerequisite for ensuring the quality of tobacco. However, in the actual production process, the complex background environment and the variability of impurity shapes can affect the accuracy of impurity detection by tobacco robots, which leads to a decrease in product quality and an increase in health risks. To address this problem, we propose a new online detection method of tobacco impurities for tobacco robot. Firstly, a BCFormer attention mechanism module is designed to effectively mitigate the interference of irrelevant information in the image and improve the network's ability to identify regions of interest. Secondly, a Dual Feature Aggregation (DFA) module is designed and added to Neck to improve the accuracy of tobacco impurities detection by augmenting the fused feature maps with deep semantic and surface location data. Finally, to address the problem that the traditional loss function cannot accurately reflect the distance between two bounding boxes, this paper proposes an optimized loss function to more accurately assess the quality of the bounding boxes. To evaluate the effectiveness of the algorithm, this paper creates a dataset specifically designed to detect tobacco impurities. Experimental results show that the algorithm performs well in identifying tobacco impurities. Our algorithm improved the mAP value by about 3.01% compared to the traditional YOLOX method. The real-time processing efficiency of the model is as high as 41 frames per second, which makes it ideal for automated inspection of tobacco production lines and effectively solves the problem of tobacco impurity detection.

## 1 Introduction

Tobacco is a critical cash crop, and the quality of its products directly impacts consumer health and satisfaction, as well as the tobacco industry's development and competitiveness. The presence of impurities such as metals, plastics, paper and plant materials can seriously affect the quality and safety of tobacco products (Girma Regassa and Chandravanshi, 2016). In this context, neural networks offer a promising solution for the effective detection and removal of these impurities. Using deep learning and computer vision techniques (Lu et al., 2022), tobacco robots can autonomously detect and classify impurities, thereby improving the quality of tobacco products.

At present, the common impurities detection methods used in the tobacco production line are mainly manual visual detection and mechanical sorting (Chao et al., 2020). Artificial visual detection has the disadvantages of low efficiency, large error, high labor intensity, etc., which is difficult to meet the requirements of high-speed production lines. Mechanical sorting refers to the use of mechanical devices for screening, blowing, adsorption and other operations to separate the impurities from the tobacco. Although this method can improve the detection speed, for the shape and size of small impurities similar to tobacco, such as metal shavings, plastic pieces, etc., it is difficult to effectively identify and remove, and the instrument is expensive, which limits its promotion and application.

In recent years, deep learning (Kumar et al., 2022; Lin et al., 2022; Zhang et al., 2023; Han et al., 2024a,b; Lakatos et al., 2024) and neural network techniques (Sun et al., 2023, 2024a,b) have flourished in the field of computer vision, and object detection methods based on these techniques have been widely used in industrial scenarios (Qi et al., 2020). Compared to traditional computer vision methods deep learning uses multiple layers of complex nonlinear mapping (Nagamine et al., 2016), these methods can learn more complex features and improve the accuracy and speed of detection. Secondly, deep learning techniques are capable of end-to-end learning (Coleman et al., 2017) and can automatically capture valid features.

The development of object detection techniques based on deep learning is mainly divided into two types: two-stage detection methods and single-stage detection methods. The two-stage detection methods are R-CNN (Girshick et al., 2014), Fast R-CNN (Girshick, 2015), Faster R-CNN (Ren et al., 2016), Cascade R-CNN (Cai and Vasconcelos, 2018) and Mask R-CNN (He et al., 2017). Based on Faster R-CNN, Ma et al. (2022) and others used the K-means algorithm to generate clustering centers based on the actual distribution characteristics of object sizes, and performed a homogenization operation on the clustering centers to generate the adaptive anchor box parameters, which improves the ability of the regional suggestion network to search for multi-scale objects. Sha et al. (2022) used a multi-level fusion structure to generate multi-scale feature maps with precise position information and semantic features, and then corrected the scale of candidate regions in RPN to improve the detection accuracy of multi-scale aircraft objects in remote sensing images. Xin et al. (2023) removed redundant deep features to improve the network accuracy and reduce the number of parameters by 38.4%. To reduce the background clutter, the CBAM attention module (Woo et al., 2018) is introduced into the backbone of the feature extraction network, which improves the model's detect ability.

For single-stage object detection, commonly used detection algorithms are the SSD (Liu et al., 2016), RetinaNet (Lin et al., 2017), YOLO series (Redmon et al., 2016), EfficientDet (Tan et al., 2020), and CornerNet (Law and Deng, 2018). Yin and Wang (2022), proposed Attention Feature Fusion SSD (AFF). First, the shallow feature information is fused using the Attention Feature Fusion module to reduce noise and improve the correlation of distant pixels in the feature map. Second, a focused classification loss function is used to solve the model degradation problem caused by the imbalance of positive and negative samples during the training process. Wenlong et al. (2024) used Swin Transformer (Liu Z. et al., 2021) as the backbone network to improve the feature extraction capability of the network. The Adaptive Contextual Feature Extraction module is proposed to adaptively adjust the sensory field using deformable convolutions with different zero rates to extract contextual features and improve the effect of multi-scale object detection. The FreeAnchor module (Wang et al., 2022) is introduced to solve the problem of dense small objects in images by designing an optimized anchor frame matching strategy from the perspective of large release estimation. Xiao-pei et al. (2023) introduced the channel-global attention mechanism (Liu Y. et al., 2021) in the backbone network to improve the feature extraction ability for objects of different scales and suppress the interference of redundant information. A dense upsampling convolution module (Sediqi and Lee, 2021) is introduced to expand low-resolution feature maps and improve the fusion effect of different feature maps.

An increasing number of scholars have undertaken research in tobacco impurity detection. To illustrate, Shaotang et al. (2009) presented a methodology for identifying foreign matter that involved constructing a standard color library and a typical foreign matter color library. They removed typical foreign matter colors and revised the standard color library. Additionally, they proposed monitoring the action of solenoid valves as a novel approach to detecting larger foreign objects. Additionally, Fuguang and Xiaoqing (2012) utilized 20 × 20 pixel blocks as the unit of measure for cigarette images. These blocks were categorized into two groups: containing foreign objects and not containing foreign objects. A clustering algorithm based on the Adaptive Iterative Self-Organizing Data Analysis Technique (ISODATA) was employed to cluster the pixel blocks that did not contain foreign objects, and subsequently to determine the clustering center. Finally, the authors converted the clustering center into HSI color space and traversed the cells in the cigarette image to detect foreign objects. Furthermore, Li et al. (2021) proposed a stacked convolutional neural network, which effectively recognized and detected moldy tobacco images by extracting and aggregating image features using convolutional kernels of varying sizes from three branches in a stepwise manner.

The following conclusions can be drawn from the above analysis:

(1) These methods deserve more processing power and memory when dealing with complex feature extraction tasks.

(2) When the object size is too small, the model will have misdetection and false detection, which will seriously affect the detection accuracy and lead to the failure of the reliable tobacco impurity detection task.

(3) Impurity detection methods are capable of detecting fewer types of impurities, resulting in an inability to effectively identify and analyse different types of impurities in complex industrial scenarios, thus affecting product quality and safety.

To address the above issues and improve the detection accuracy of tobacco impurities, a new online detection method of tobacco impurities for tobacco robot is proposed in this article. The main innovation points of this article are as follows:

(1) A BCFormer module is proposed to improve the network's ability to detect regions of interest by weakening non-critical information in the image.

(2) An DFA module is proposed to enhance the fusion of detail information from shallow feature maps and semantic information from deep feature maps.

(3) An optimized loss function is proposed to provide more feature information and reduce the inference time during the training phase, thereby improving the accuracy and robustness of the detection algorithm against tobacco impurities.

(4) In this paper, images of tobacco impurities in different weather conditions have been collected to create a tobacco impurity dataset.

## 2 Methods

### 2.1 Overall network structure

**Figure 1:**
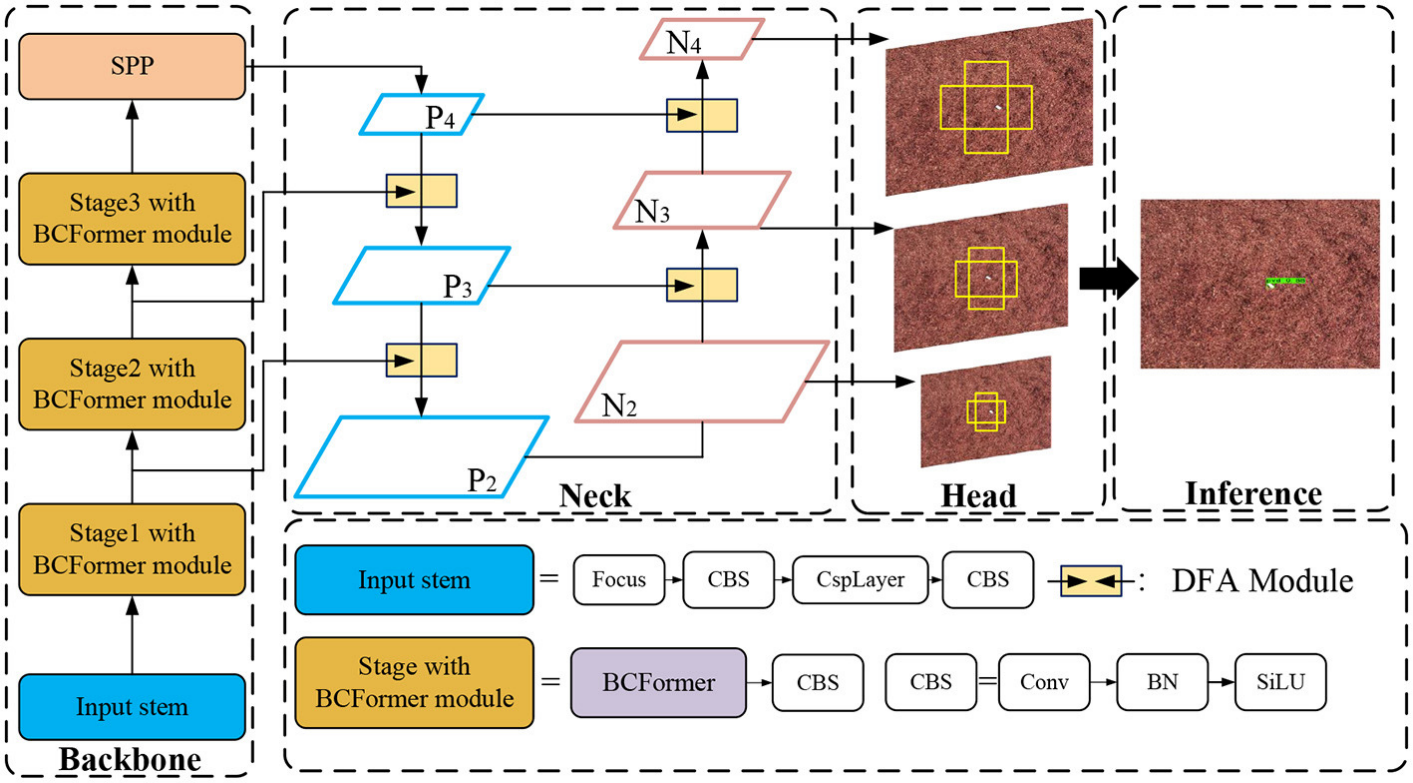
Overall structure of the network.

Figure 1 illustrates the structure of our network. The network uses YOLOX as a base model for detecting tobacco impurities in complex environments. Firstly, we added the BCFormer attention mechanism to the backbone network to extract key feature information of the object region by learning the importance of each feature. Secondly, the Dual Feature Aggregation module (DFA) module is added to the Neck part to better achieve the fusion of the low-level detail information with the high-level semantic information, so as to reduce the interference of the complex background on the detection. Finally, the GIoU loss function is used as a regression loss function to enable the model to learn more accurate bounding box predictions, further improving the accuracy of object detection.

### 2.2 BCFormer module

Attention is an important mechanism that focuses on highlighting important information and adjusting weights to improve model performance. It is widely used in various object detection tasks to improve the accuracy and robustness of detection by selecting and focusing on regions or features that are relevant to the object. In general, the attention mechanism, usually referred to as self-attention, is a key technique in the Transformer model. It assigns weights by calculating the dependencies between different positions in the input sequence, thus adjusting the level of attention to each position in the sequence. However, in the original Transformer network, this structure requires the computation of the correlation between any two elements in the input sequence. The computational complexity grows exponentially with the length of the sequence, so models using such techniques require a large amount of computational resources.

**Figure 2:**
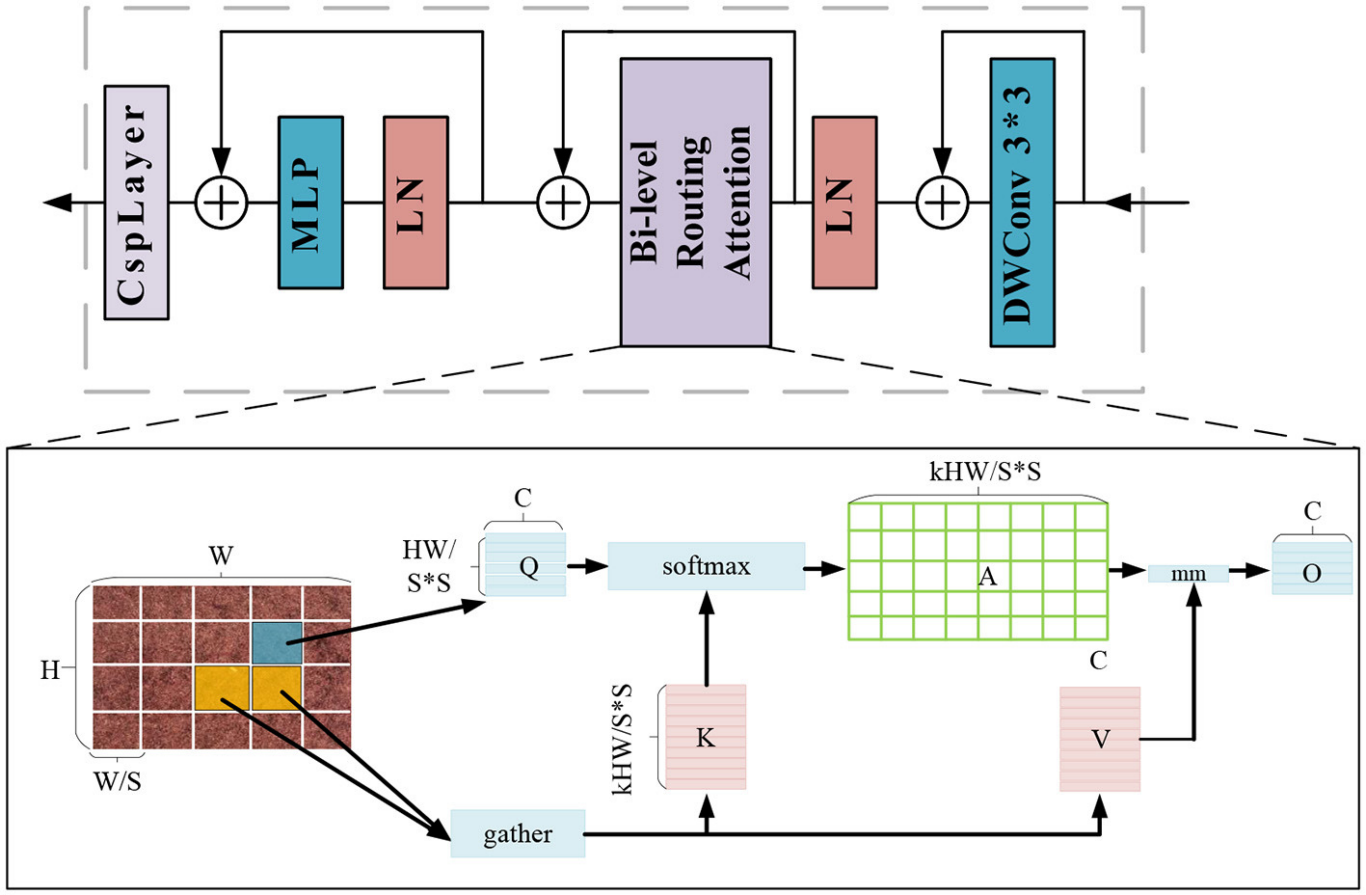
Network structure of BCFormer.

To solve the above problem, we propose a visual transformer model called BCFormer, which introduces a Bi-Level Routing Attention (BRA) module in the visual transformer. This module improves the feature representation by enabling information interaction between global and local attention levels. This is because the global attention mechanism captures the overall structure and global information of the image, while the local attention mechanism captures the details and local features of the image. Therefore, the introduction of the Bi-Level Routing Attention (BRA) module can better deal with the global and local relationships in the image and effectively capture the structural and detailed features of the image. This model significantly improves the performance in the detection task, and its structure is shown in Figure 2.

In this paper, Bi-Level Routing Attention is used as the basic building block, so the BCFormer module first uses 3 × 3 convolution to implicitly encode the relative position information, and then performs the normalization operation by Layer Normalization (LN). Then, after the BRA (Bi-Level Routing Attention) module, the feature map is divided into S × S non-overlapping regions, and most of the irrelevant key-value pairs are filtered out from the rough regions, and only a small portion of the routing regions are retained. Finally, a multilayer perceptron (MLP) and CspLayer are used to select the most appropriate weights and biases for feature transformation, information reorganization and feature extraction.

In the BRA module we define several key concepts: Q is used to compute the weighted relevance of a query with respect to a keyword, K denotes the keyword or identifier that provides information or is used for matching purposes, V is the value associated with the query result and the keyword information, C is a scalar factor used to adjust the allocation of attention and control the focus of attention, A is the adjacency matrix used to represent the semantics between two regions of correlation, O is the output of the attention mechanism.

### 2.3 Dual Feature Aggregation module

There are objects of different sizes in the image, and objects of different sizes have different features. Simple objects can be discriminated using shallow features, while complex objects can be discriminated using deep features. Previous studies have shown that shallow features are able to extract more information such as location and details due to their high resolution, but lack semantic information and are susceptible to noise; on the contrary, deep features contain more semantic information but have lower resolution.

The YOLO algorithm will gradually lose some of the image's feature information as the network layers deepen during the detection process, resulting in insufficient feature learning for small objects and poor detection performance. Therefore, some researchers try to adjust the feature maps to the same size and then merge them. However, this method may lose some information of the feature map itself during the merging process.

**Figure 3:**
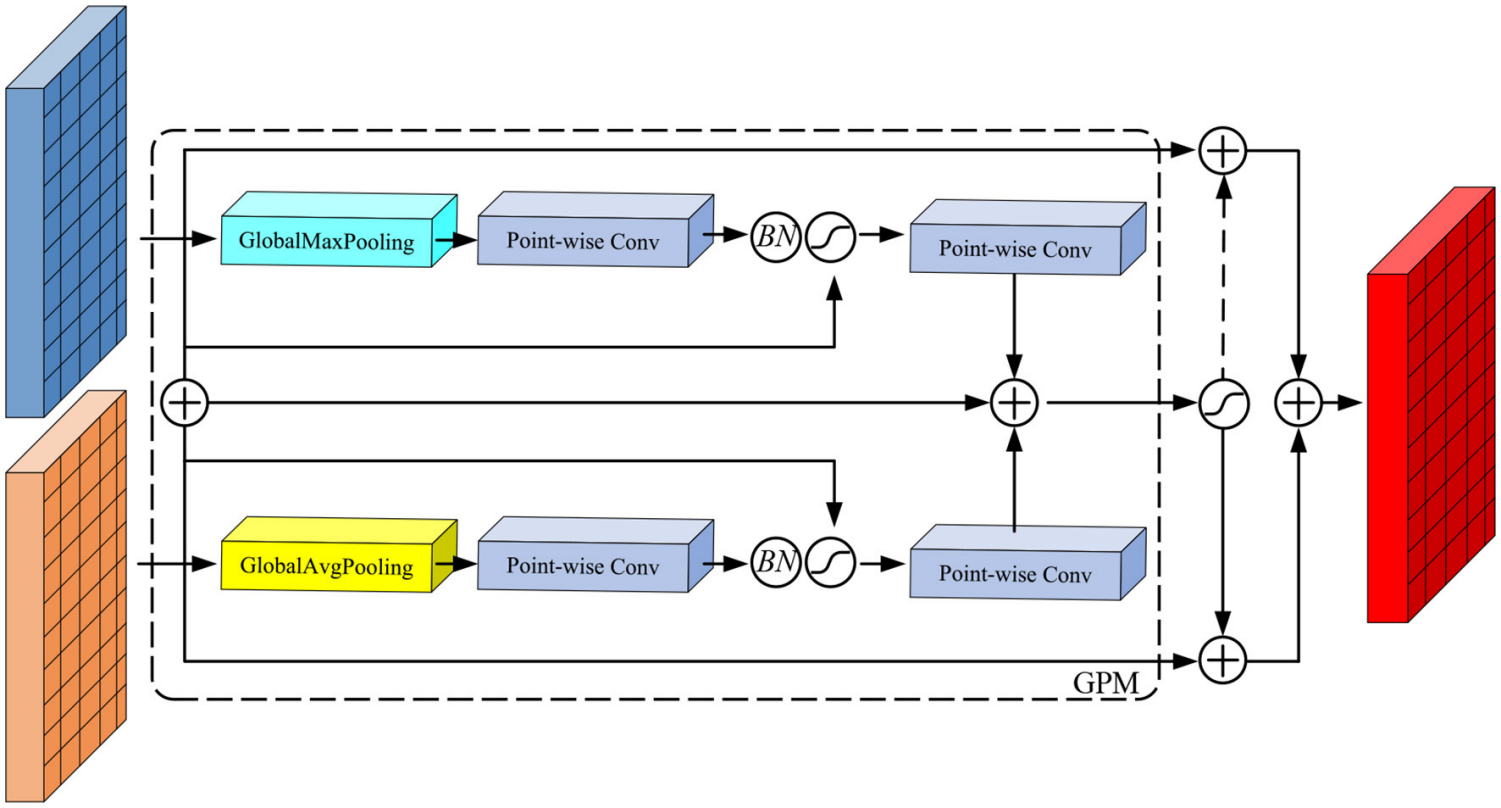
Network structure of DFA module.

To address this problem, we propose the Dual Feature Aggregation (DFA) module, shown in Figure 3, which aims to improve the information fusion between feature maps at different scales. The specific workflow of this module is as follows: first, we perform a simple alignment fusion process on the feature tensor of two branch networks for subsequent processing. Next, the fusion results are used as guidance information to generate weight parameters by the GPM module. Finally, these weights are used to perform a weighted transformation on the fused feature tensor, and the weighted features are used as inputs to the next module.

Since the DFA module cannot be directly integrated into the structure of YOLOX, we have modified it to suit YOLOX's needs. The specific structure is shown in Figure 3. We apply the DFA module structure to replace the Concat operation in Neck to establish an attention mechanism between Input1 and Input2. DFA module is not only able to fuse the information of feature maps of different scales, but also able to learn to establish the attention to feature maps of different scales, which improves the accuracy of the network in detecting tobacco impurities.

### 2.4 Loss function

The loss function of YOLOX mainly includes regression loss, confidence loss and classification loss. Among them, the cross-entropy loss function is used for confidence loss and classification loss, while the IoU loss function is used for regression loss.

In regression loss, the IoU loss function is used to calculate the degree of overlap between the predicted frame and the real frame, but it cannot reflect the distance between the two frames, resulting in a gradient of 0, which cannot be optimized. To solve this problem, this paper uses GIoU instead of IoU as the loss function. The GIoU is shown in Equations 1 and 2:


(1) $$\begin{array}{l} GIoU=IoU-\frac{\left|C\backslash \left(A\cup B\right)\right|}{\left|C\right|} \end{array}$$

(2) $$\begin{array}{l} I\text{oU}=\frac{A\cap B}{A\cup B} \end{array}$$

**Figure 4:**
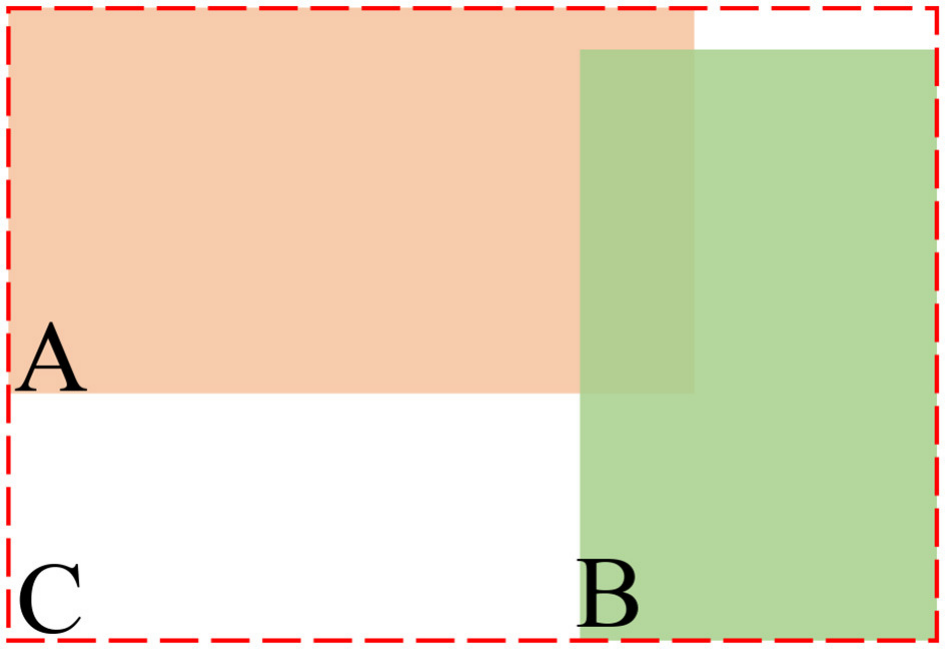
Illustration of GIoU loss formula.

Where A represents the prediction frame, B represents the true frame, and C represents the A and B minimal outer box, as shown in Figure 4.

When YOLOX adopts the GIoU loss as the regression loss function, the function integrates the overlap area and the scale, which better satisfies non-negativity and symmetry during training and improves detection accuracy. The *L*_*GIoU*_ formula is shown in Equation 3:


(3) $$\begin{array}{l} L_{GIoU}=1-GIoU \end{array}$$

Therefore, the loss function of this model is specified is shown in Equation 4:


(4) $$\begin{array}{l} Loss=\frac{L_{cls}+L_{GIoU}+\lambda L_{conf}}{N_{pos}} \end{array}$$

Where λ is the regression loss compensation coefficient, which in this paper is assumed to be 5. *N*_*pos*_ is the number of anchor points divided into positive samples, and confidence loss and classification loss are shown in Equation 5:


(5) $$BCE=-\log(P_{t})=\{\begin{array}{l} -\log(y),(y=1)\\ -\log(1-y),(y=0) \end{array}$$

Where *y* = 1 is a positive sample and *y* = 0 is a negative sample.

By introducing the loss function into the constructed network, the convergence speed of the model in the training process is effectively improved, which in turn further improves the accuracy of the model for detecting tobacco impurities.

## 3 Experiments

### 3.1 Dataset

**Figure 5:**
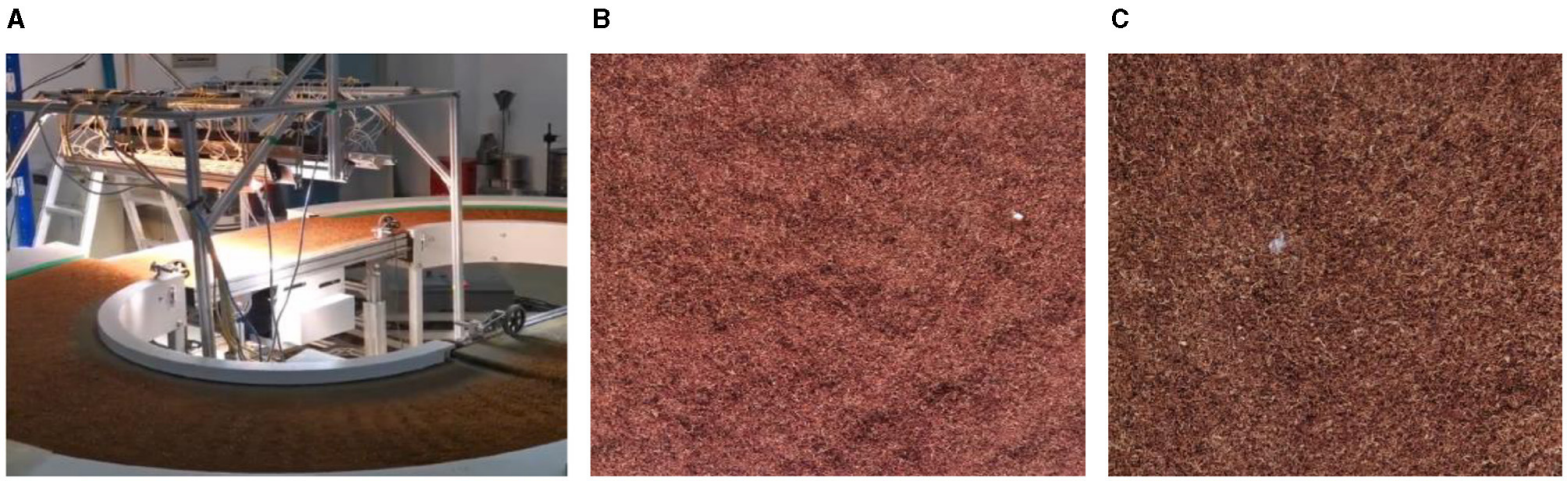
Tobacco impurities image acquisition platform and image. **(A)** Tobacco debris image acquisition platform. **(B)** Images collected during the day. **(C)** Images collected at night.

To verify the effectiveness of the algorithm in detecting tobacco impurities, a dataset of tobacco impurities was constructed in this paper. The images of this dataset were taken by researchers from the Zhengzhou Tobacco Research Institute of China Tobacco Corporation on a real production line in two scenarios: day and night, as shown in Figure 5.

The total dataset contains 2,454 images, including 200 fabrics, 250 sponges, 100 sawdust, 150 belts, 200 PV bands, 256 latex, 344 plastics, 150 plastic films, 100 leaves, 150 filaments, 100 wire tubes, 200 rubber, 50 feathers and 204 paper scraps, among other common tobacco impurities categories. In the dataset, 2,000 images were selected as the training set and the remaining images were used as the validation set. The specific data classification is shown in Table 1.

**Table 1:** Introduction to the dataset.

**Name**	**Categories**	**Number of sheets**
bm	Fabric	200
hm	Sponge	250
mp	Sawdust	100
pd	Belt	150
pvd	PV band	200
rj	Latex	256
sl	Plastic	344
slbm	Plastic film	150
sy	Leaf	100
szw	Filament	150
xg	Wire tube	100
xj	Rubber	200
ym	Feather	50
zp	Scraps of paper	204

**Figure 6:**
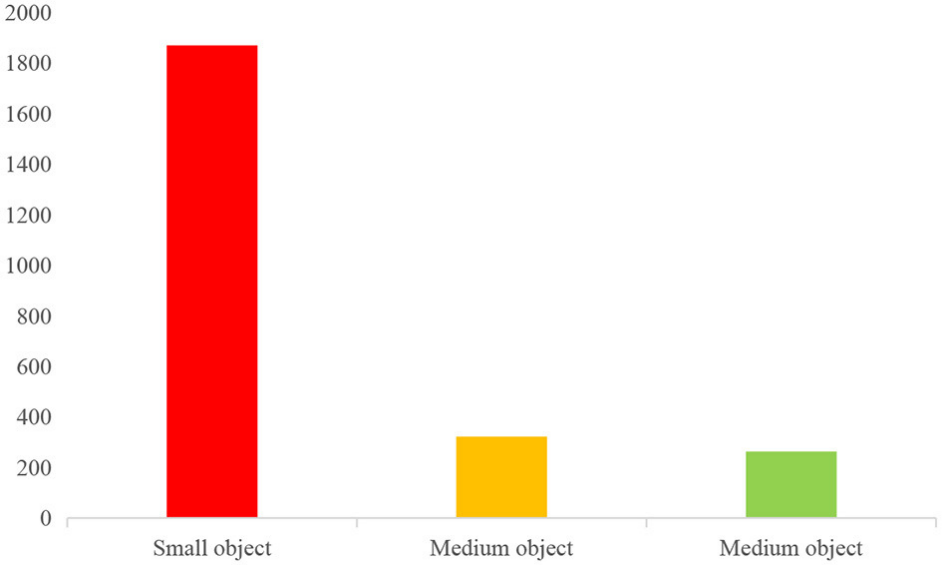
Object class size allocation.

In this dataset, the object area is <32 × 32 pixel, we consider it a small object; when the object area is between 32 × 32 pixels and 96 × 96 pixels, it is considered as a medium object; and when >96 × 96 pixels, it is considered as a large object. In this case, we can classify small, medium and large objects based on the area ratio of the label box and the image. After classification, the number of small, medium and large objects in our dataset is 1,869, 322 and 263, respectively. The specific sample distribution for each category and the overall sample distribution are shown in Figure 6.

### 3.2 Implementation details

The experimental environment of this paper uses a computer with Windows 10, 64-bit operating system, Intel(R) Core(TM) i7-11700 processor, NVIDIA RTX4000 discrete graphics card, and 8G of RAM. It runs under Python 3.6, Pytorch 1.7.0 and CUDA 11.8.

In this work, a freeze and thaw procedure is used in the training phase. At the beginning of training, for the first 50 generations, a batch size of 16 and a learning rate of 1e-3 are used for freeze training. From 50 to 300 generations, a batch size of 8 and a learning rate of 1e-4 is used for thaw training. The optimizer used was adam with an internal dynamics parameter of 0.937. Due to the limitations of the mAP calculation principle, the network has to obtain almost all prediction frames when calculating the mAP. Therefore, the confidence level was set at 0.5. Note that all images were automatically scaled to 640 × 640 for the detection task.

### 3.3 Evaluation metrics

In this experiment, Precision (P), Recall (R), Mean Average Precision (mAP) and FPS are used as evaluation indices. P, R, and mAP are calculated as shown in Equations 6–9:


(6) $$\begin{array}{l} P=\frac{TP}{TP+FP} \end{array}$$

(7) $$\begin{array}{l} R=\frac{TP}{TP+FN} \end{array}$$

(8) $$AP=\int_{0}^{1}P(R)dR$$

And mAP is the mean of all APs in N classes.


(9) $$mAP=\frac{\sum_{k=1}^{n}AP_{k}}{n}$$

Where TP are positive samples predicted as positive samples, FP are negative samples predicted as positive samples, FN are positive samples predicted as negative samples, $AP_{\overset{•}{\kappa }}$ refers to the average precision of the kth class of detected objects, i.e. the area under the PR curve, and mAP is the sum of the average precision of all classes divided by the number of classes.

### 3.4 The ablation experiment

In this paper we present a series of comparative experiments, each designed to evaluate the effectiveness of the proposed modules. Table 2 shows the results of the removal experiments performed on the Tobacco Impurities dataset, with the bold graph showing the best results. Our results show that the addition of the BCFormer module leads to a 0.16% improvement in P, a 0.37% improvement in R and a 0.93% improvement in mAP, despite the reduction in FPS. This suggests that the BCFormer module improves the focus of the network on relevant information and suppresses irrelevant data. Replacing the Concat section in Neck with the DFA module resulted in a 4.2% improvement in R and a 2.19% improvement in mAP, with a decrease in FPS. The applicability of the DFA module for tobacco impurity detection is demonstrated. In addition, the inclusion of the GIoU loss improved P by 1.89%, R by 3.63% and mAP by 2.19%, demonstrating the effectiveness of this module in the detection of tobacco impurities. Finally, the BCFormer, DFA and GIoU losses were combined to give YOLOX+BCFormer+DFA+GIoU. The experimental results showed that this model increased precision, recall and mean average precision by 0.91%, 9.86%, and 3.01% respectively.

**Table 2:** The results of the ablation experiments.

**YOLOX**	**BCFormer**	**DFA**	**GIoU**	**P (%)**	**R (%)**	**mAP (%)**	**FPS**
√	–	–	–	96.76	87.20	95.50	**51.36**
√	√	–	–	96.92	87.57	96.43	44.15
√	–	√		96.34	91.40	97.69	44.53
√	–	–	√	98.65	90.83	97.69	48.61
√	√	√	√	**97.67**	**97.06**	**98.51**	41.51

Table 2 displays the results of the ablation experiments that were conducted on tobacco Impurities data using this method.

### 3.5 Comparison with existing methods

To demonstrate the effectiveness of the method in detecting tobacco impurities, the same trained parameters were used to compare with other state-of-the-art methods on the same tobacco impurity dataset. Table 3 compares the mAP, FPS, F1, and Params obtained by different algorithms, with the bold graph showing the best results.

**Table 3:** A comparison of this method with other methods.

	**SSD**	**YOLOv3**	**Faster-RCNN**	**Centernet**	**YOLOv5**	**YOLOv7**	**YOLOX**	**Ours**
bm	73.82	77.23	76.62	75.65	72.95	89.21	91.59	**99.12**
hm	100.00	100.00	100.00	100.00	100.00	100.00	100.00	**100.00**
mp	100.00	99.65	97.52	98.75	100.00	100.00	100.00	**100.00**
pd	40.22	41.32	40.39	39.21	38.34	77.32	78.95	**93.50**
pvd	74.25	75.65	76.52	77.59	78.51	94.25	93.47	**100.00**
rj	8.22	11.35	12.32	10.25	9.52	100.00	100.00	**98.21**
sl	94.32	98.22	98.56	100.00	100.00	100.00	100.00	**100.00**
slbm	87.55	90.21	89.21	93.21	96.70	99.62	99.05	**100.00**
sy	88.87	84.21	82.12	82.36	88.45	88.41	88.40	**98.11**
szw	90.11	92.11	89.39	88.17	89.91	95.26	94.26	**98.16**
xg	99.12	98.89	99.36	98.52	100.00	100.00	100.00	**100.00**
xj	88.34	90.21	91.36	88.19	89.21	88.32	91.90	**92.06**
ym	100.00	100.00	100.00	99.17	100.00	100.00	100.00	**100.00**
zp	100.00	99.54	99.91	99.34	100.00	98.22	100.00	**100.00**
mAP	81.77	82.76	82.38	82.17	83.11	95.04	95.50	**98.51**
FPS	42.84	49.82	47.32	42.24	46.17	50.29	**51.36**	41.51
F1	0.92	0.95	0.96	0.95	0.93	0.91	0.96	**0.97**
Params	26.28	**61.94**	28.48	32.70	31.07	37.62	9.12	9.98

From Table 3 it can be concluded that the mAP and F1 values obtained by this algorithm are better than the other algorithms and have a smaller number of parameters. Among the 14 types of impurities detected, nine of them achieved 100% mAP detection. Compared with other types of impurities, the detection accuracy of xj is relatively low, mainly due to the severe occlusion of xj, which makes it difficult to extract effective feature information. However, in our method, the real-time detection performance of the model is affected to some extent, as the number of parameters increases while the object detection accuracy improves.

**Figure 7:**
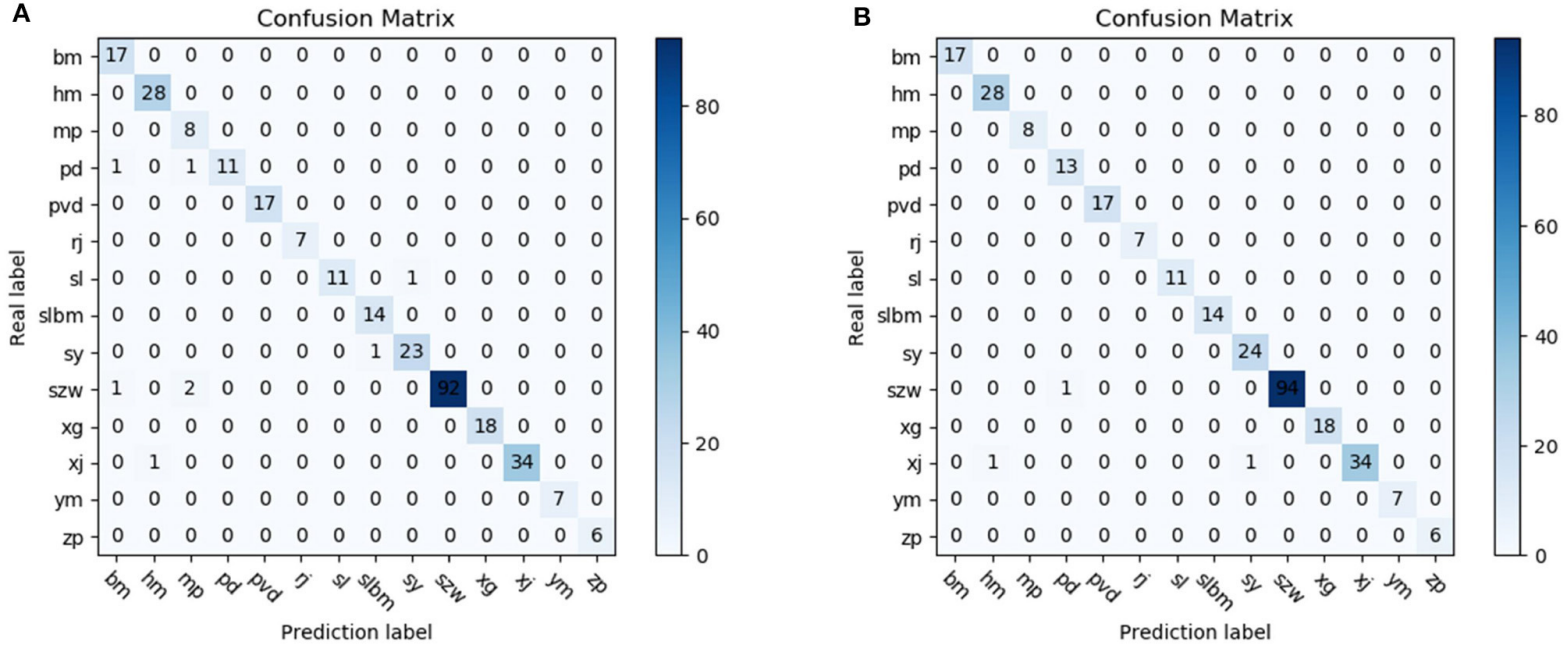
Confusion matrix for tobacco impurities detection. **(A)** YOLOX. **(B)** Ours.

According to Figure 7, we can find that 17, 28, 8, 13, 17, 7, 11, 14, 24, 94, 18, 34, 7, 6 were detected for cloth, sponge, wood chip, belt, pv belt, latex, plastic, plastic film, leaf, filament, wire pipe, rubber, feather and paper, respectively, which is more than the original model, with two more belts, one more leaves, and two more filaments detected. The experimental data proved the correctness of the method proposed in this paper, and provided a certain reference significance for the improvement of the detection technology of tobacco filament impurities.

**Figure 8:**
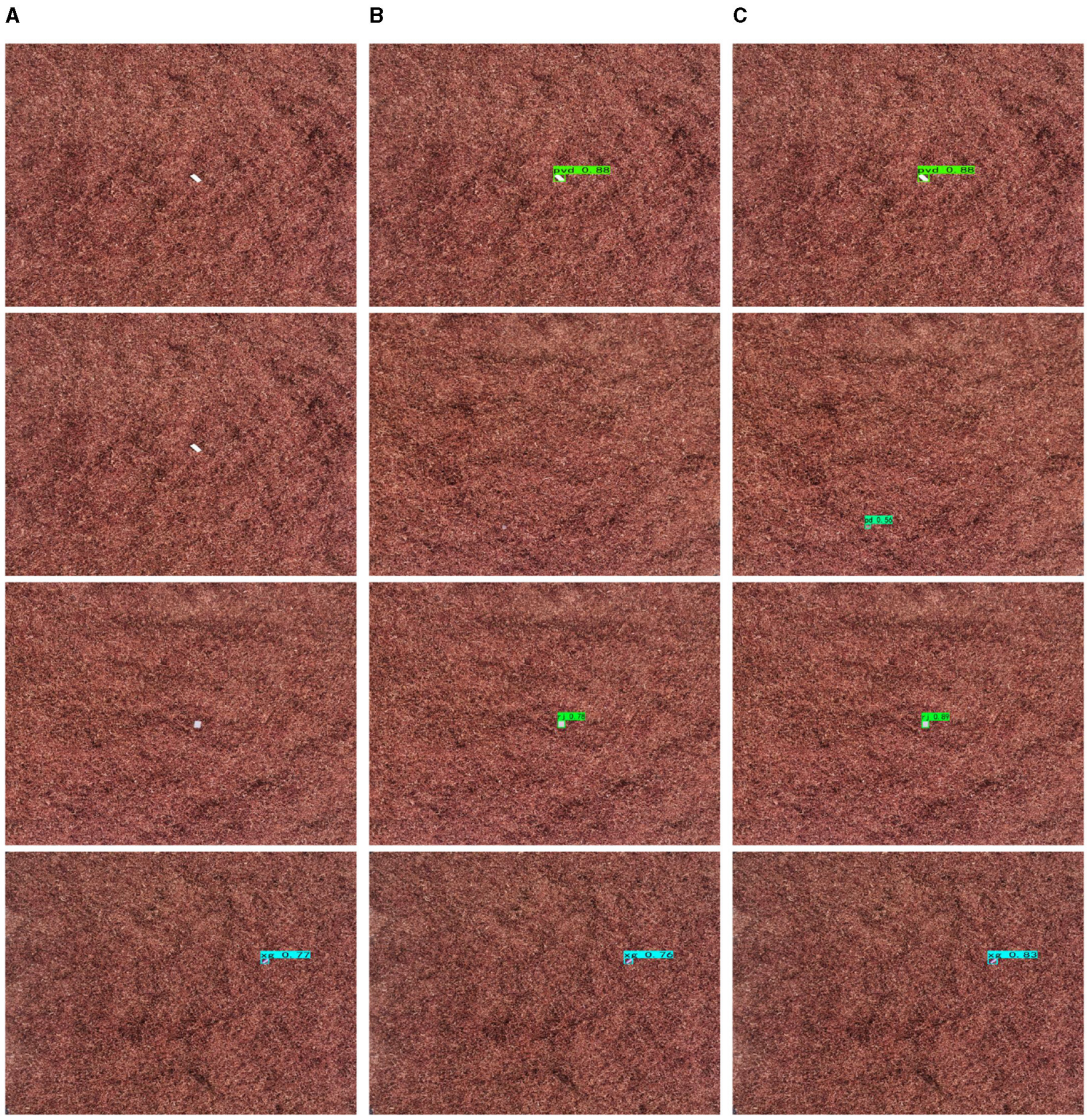
Comparison of images of tobacco impurities. **(A)** YOLOv5. **(B)** YOLOX. **(C)** Ours.

Figure 8 shows the detection results of four types of impurities, namely pvd, pd, xj, and xg, in daytime scenarios. It can be seen that compared with the other two state-of-the-art algorithms, the algorithm proposed in this paper not only effectively detects impurities, but also has a relatively high confidence level. As can be seen, the addition of the BCFormer module to extract multi-scale feature information can significantly improve the sensory field of the network. In addition, incorporating the DFA module can enable the network to focus more precisely on the object region of interest, thereby improving the object detection accuracy. Thus, it is evident that the method proposed in this paper is capable of significantly reducing the leakage object detection rate under daytime conditions.

**Figure 9:**
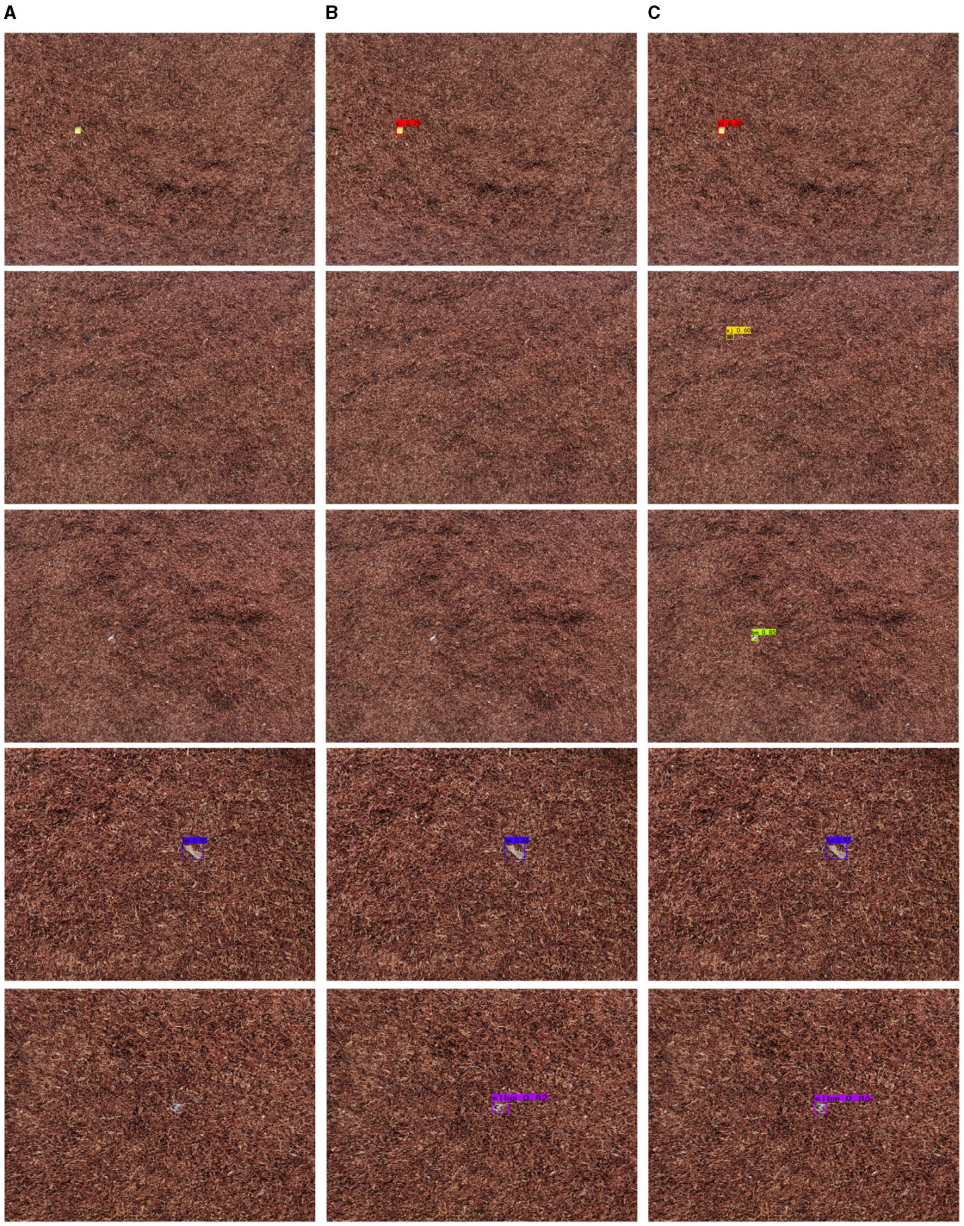
Comparison of images of tobacco impurities. **(A)** YOLOv5. **(B)** YOLOX. **(C)** Ours.

The detection results of different algorithms for impurities such as bm in the night environment are shown in Figure 9. Similar conclusions can be drawn that our method can achieve excellent impurity detection results, the clutter detection level of which is higher than that of the comparison methods. In addition, this algorithm can also achieve excellent detection performance for impurities with strong occlusion.

In summary, this algorithm can achieve accurate impurity detection in all weather conditions, providing accurate data for accurate impurity removal in subsequent tobacco production lines.

**Figure 10:**
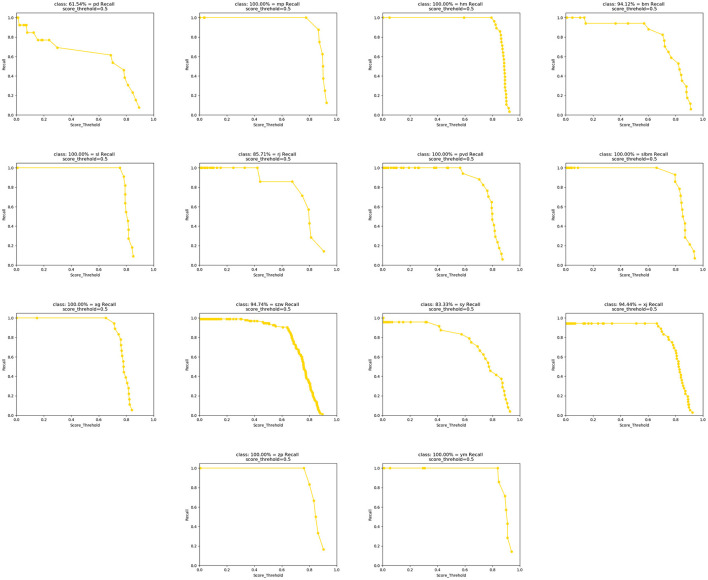
Fourteen types of recalls obtained.

Figure 10 shows the recall detection results of 14 types of impurities obtained by the algorithm in this article. It can be seen that the algorithm in this article can achieve a high recall rate for most impurities, but the detection effect for belts is poor. After analysis, it was found that the shape volume of belts is small and their morphological features are similar to those of tobacco, which further affects the accuracy of belt feature extraction.

### 3.6 Practical scenario application

**Figure 11:**
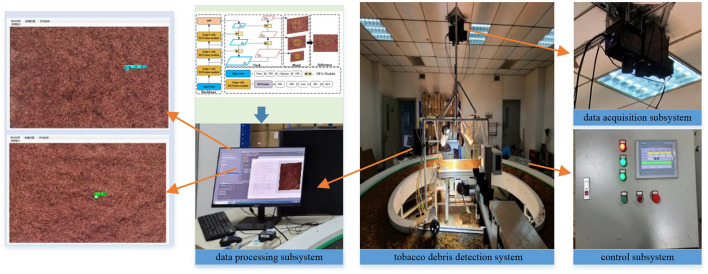
Robotic system for tobacco impurity detection.

According to the algorithm proposed in this paper, a tobacco impurity detection system is designed and applied to a tobacco impurity detection robot, which mainly consists of an image data acquisition subsystem and a data processing subsystem, as shown in Figure 11.

**Figure 12:**
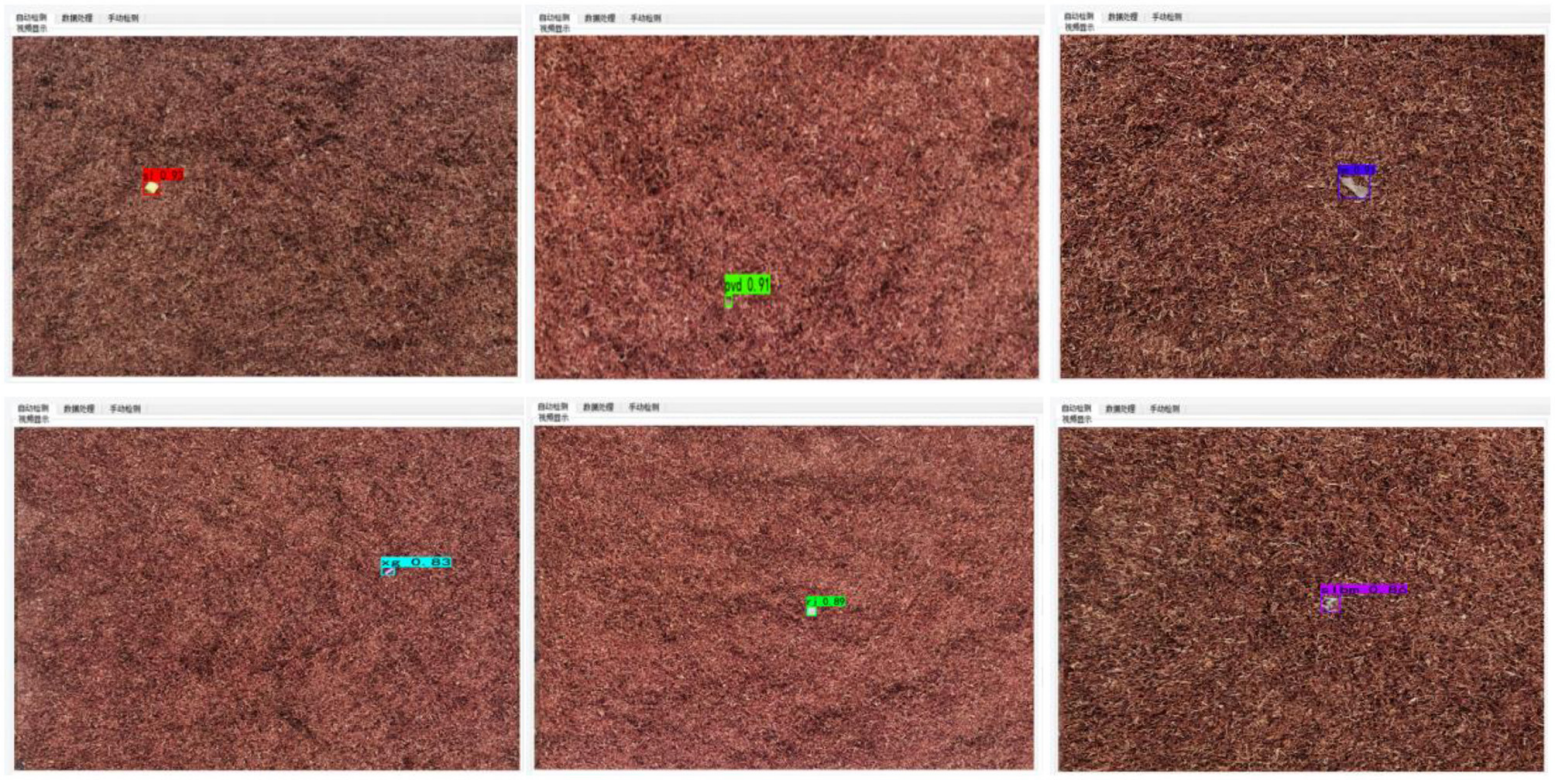
Detection effect of tobacco impurities on the production line.

Figure 12 shows the detection effect of tobacco impurities on the actual production line. It can be seen that the system can achieve accurate detection of various impurities, and the average confidence level is more than 0.9, which fully verifies the effectiveness of the algorithm in this paper. At present, the system has been put into operation and is in good working condition. The accuracy of tobacco impurity detection is high, effectively improving the intelligent level of tobacco impurity detection.

## 4 Conclusions

To improve the detection accuracy of tobacco impurities, this paper proposes a new online detection method of tobacco impurities for tobacco robot, which significantly improves the accuracy of tobacco impurity detection. The YOLOX-based algorithm with BCFormer module and DFA module enables the tobacco robot to pay more attention to the region of interest and effectively suppress irrelevant information in the image. The GIoU loss function further reduces the model complexity and improves the detection efficiency. The tobacco robot based on this proposed method, implemented in a real production line, performs well and achieves accurate detection of impurities with high confidence. However, there are still some challenges in detecting leakage in complex contexts. To further improve the efficiency of this method, we will strive to optimize it in our next research to reduce the leakage detection rate in complex environments.

In future work, we will continue to collect images of more types of tobacco impurities to improve the model to detect a larger number of impurities. Meanwhile, we will also improve and optimize the model according to detection tasks in other scenarios to extend the applicability of the model in this paper.

## Data availability statement

The data analyzed in this study is subject to the following licenses/restrictions. Requests to access these datasets should be directed to zzuli_407@163.com.

## Author contributions

LZ: Writing – original draft, Writing – review & editing. DL: Writing – original draft, Writing – review & editing. DX: Writing – original draft. EZ: Writing – review & editing. ZL: Writing – review & editing. JL: Writing – review & editing. JD: Writing – review & editing. SL: Writing – original draft, Writing – review & editing.

## References

[B1] Cai Z. Vasconcelos N. (2018). “Cascade R-CNN: delving into high quality object detection,” in Proceedings of the IEEE Conference on Computer Vision and Pattern Recognition (Salt Lake City, UT: IEEE), 6154–6162. 10.1109/CVPR.2018.00644

[B2] Chao M. Kai C. Zhiwei Z. (2020). Research on tobacco foreign body detection device based on machine vision. Trans. Inst. Meas. Control. 42, 2857–2871. 10.1177/0142331220929816

[B3] Coleman C. Narayanan D. Kang D. Zhao T. Zhang J. Nardi L.. (2017). Dawnbench: an end-to-end deep learning benchmark and competition. Training 100:102.

[B4] Fuguang Y. Xiaoqing W. (2012). Research on online recognition algorithm of tobacco foreign body based on adaptive clustering and color difference analysis. J. Chongqing Educ. Inst. 25, 27–30.

[B5] Girma Regassa G. R. Chandravanshi B. (2016). Levels of heavy metals in the raw and processed Ethiopian tobacco leaves. Springerplus 5:232. 10.1186/s40064-016-1770-z 27026926 PMC4771704

[B6] Girshick R. (2015). “Fast R-CNN,” in Proceedings of the IEEE International Conference on Computer Vision (Santiago: IEEE), 1440–1448. 10.1109/ICCV.2015.169

[B7] Girshick R. Donahue J. Darrell T. Malik J. (2014). “Rich feature hierarchies for accurate object detection and semantic segmentation,” in Proceedings of the IEEE conference on computer vision and pattern recognition (Columbus, OH: IEEE), 580–587. 10.1109/CVPR.2014.81

[B8] Han C. Liang J. C. Wang Q. Rabbani M. Dianat S. Rao R.. (2024a). Image translation as diffusion visual programmers. arXiv [Preprint]. arXiv:2401.09742. 10.48550/arXiv.2401.09742

[B9] Han C. Wang Q. Cui Y. Wang W. Huang L. Qi S.. (2024b). Facing the elephant in the room: visual prompt tuning or full finetuning? *arXiv* [Preprint]. arXiv:2401.12902. 10.48550/arXiv.2401.12902

[B10] He K. Gkioxari G. Dollár P. Girshick R. (2017). “Mask R-CNN,” in Proceedings of the IEEE international conference on computer vision (Venice: IEEE), 2961–2969. 10.1109/ICCV.2017.322

[B11] Kumar B. M. Rao K. R. K. Nagaraj P. Sudar K. M. Muneeswaran V. (2022). “Tobacco plant disease detection and classification using deep convolutional neural networks,” in 2022 International Conference on Sustainable Computing and Data Communication Systems (ICSCDS) (Erode: IEEE), 490–495.

[B12] Lakatos R. Pollner P. Hajdu A. Joó T. (2024). A multimodal deep learning architecture for smoking detection with a small data approach. Front. Artif. Intell. 7:1326050. 10.3389/frai.2024.1326050 38481821 PMC10936563

[B13] Law H. Deng J. (2018). “Cornernet: detecting objects as paired keypoints,” in Proceedings of the European conference on computer vision (ECCV) (Cham: Springer), 734–750. 10.1007/978-3-030-01264-9_45

[B14] Li Y. Yun L. Ye Z. Wang K. Zhai N. (2021). Research on moldy tobacco leaf image recognition method based on convolutional neural network. Comput. Eng. Sci. 43. 10.1109/ICPECA51329.2021.9362689

[B15] Lin J. Chen Y. Pan R. Cao T. Cai J. Yu D.. (2022). Camffnet: a novel convolutional neural network model for tobacco disease image recognition. Comput. Electron. Agric. 202:107390. 10.1016/j.compag.2022.107390

[B16] Lin T.-Y. Goyal P. Girshick R. He K. Dollár P. (2017). “Focal loss for dense object detection,” in Proceedings of the IEEE international conference on computer vision (Venice: IEEE), 2980–2988. 10.1109/ICCV.2017.324

[B17] Liu W. Anguelov D. Erhan D. Szegedy C. Reed S. Fu C.-Y.. (2016). “SSD: single shot multibox detector,” in Computer Vision-ECCV 2016: 14th European Conference, Amsterdam, The Netherlands, October 11-14, 2016, Proceedings, Part I 14 (Cham: Springer), 21–37. 10.1007/978-3-319-46448-0_2

[B18] Liu Y. Shao Z. Hoffmann N. (2021). Global attention mechanism: retain information to enhance channel-spatial interactions. arXiv [Preprint]. arXiv:2112.05561. 10.48550/arXiv.2112.05561

[B19] Liu Z. Lin Y. Cao Y. Hu H. Wei Y. Zhang Z.. (2021). “Swin transformer: Hierarchical vision transformer using shifted windows,” in Proceedings of the IEEE/CVF international conference on computer vision (Montreal, QC: IEEE), 10012–10022. 10.1109/ICCV48922.2021.00986

[B20] Lu M. Jiang S. Wang C. Chen D. Chen T. (2022). Tobacco leaf grading based on deep convolutional neural networks and machine vision. J. ASABE 65, 11–22. 10.13031/ja.14537

[B21] Ma Y. Shan Y. Yuan J. (2022). Remote sensing object detection algorithm based on improved faster R CNN. Mod. Electron. Technol. 45, 58–63. 10.16652/j.issn.1004-373x.2022.03.012

[B22] Nagamine T. Seltzer M. L. Mesgarani N. (2016). “On the role of nonlinear transformations in deep neural network acoustic models,” in Interspeech, 803–807. 10.21437/Interspeech.2016-1406

[B23] Qi S. Yang J. Zhong Z. (2020). “A review on industrial surface defect detection based on deep learning technology,” in Proceedings of the 2020 3rd International Conference on Machine Learning and Machine Intelligence (New York, NY: ACM), 24–30. 10.1145/3426826.3426832

[B24] Redmon J. Divvala S. Girshick R. Farhadi A. (2016). “You only look once: unified, real-time object detection,” in Proceedings of the IEEE Conference on Computer Vision and Pattern Recognition (Las Vegas, NV: IEEE), 779–788. 10.1109/CVPR.2016.91

[B25] Ren S. He K. Girshick R. Sun J. (2016). Faster R-CNN: towards real-time object detection with region proposal networks. IEEE Trans. Pattern Anal. Mach. Intell. 39, 1137–1149. 10.1109/TPAMI.2016.2577031 27295650

[B26] Sediqi K. M. Lee H. J. (2021). A novel upsampling and context convolution for image semantic segmentation. Sensors 21:2170. 10.3390/s21062170 33804591 PMC8003770

[B27] Sha M. Li Y. Li A. (2022). Improved faster R-CNN for aircraft object detection in remote sensing images. Nat. Remote Sens. Bull 26, 1624–1635.

[B28] Shaotang Z. Dechun D. Youjun R. Puling D. Dejian K. (2009). Typical foreign body disposal methods in tobacco foreign body removal system. Tobacco Technol. 22–25.

[B29] Sun J. Li C. Wang Z. Wang Y. (2023). A memristive fully connect neural network and application of medical image encryption based on central diffusion algorithm. IEEE Trans. Ind. Inform. 10.1109/TII.2023.3312405

[B30] Sun J. Yue Y. Wang Y. Wang Y. (2024a). Memristor-based operant conditioning neural network with blocking and competition effects. IEEE Trans. Ind. Inform. 10.1109/TII.2024.3393975

[B31] Sun J. Zhai Y. Liu P. Wang Y. (2024b). Memristor-based neural network circuit of associative memory with overshadowing and emotion congruent effect. IEEE Trans. Neural Netw. Learn. Syst. 10.1109/TNNLS.2023.3348553 38194385

[B32] Tan M. Pang R. Le Q. V. (2020). “Efficientdet: scalable and efficient object detection,” in Proceedings of the IEEE/CVF Conference on Computer Vision and Pattern Recognition (Seattle, WA: IEEE), 10781–10790. 10.1109/CVPR42600.2020.01079

[B33] Wang F. Hu H. Zhou Q. Wang R. (2022). “DACFA-det: a domain adaptive calibrated free anchor detection network for agricultural similar pests,” in International Conference on Computer Graphics, Artificial Intelligence, and Data Processing (ICCAID 2021), Vol. 12168 (SPIE), 423–427. 10.1117/12.2631167

[B34] Wenlong L. Kuerban A. Yixiao C. Xu Y. (2024). Acfem-retinanet algorithm for object detection in remote sensing images. Comput. Eng. Appl. 60, 245–253. 10.3778/j.issn.1002-8331.2208-0240

[B35] Woo S. Park J. Lee J.-Y. Kweon I. S. (2018). “CBAM: convolutional block attention module,” in Proceedings of the European conference on computer vision (ECCV) (Cham: Springer), 3–19. 10.1007/978-3-030-01234-2_1

[B36] Xiao-pei L. Yin-bao Z. Yanpei L. Yun-xing Y. (2023). An improved algorithm for infrared image target detection based on YOLOV5s. Laser Infrared 53, 1043–1051. 10.13336/j.1003-6520.hve.20230816

[B37] Xin Y. Qiong W. Yazhou Y. Zhenmin T. (2023). Improved aircraft detection for optical remote sensing images based on faster r-cnn. Laser Optoelectron. Prog. 60:1228007. 10.3788/LOP221679

[B38] Yin F. Wang T. (2022). Attention feature fusion ssd algorithm for object detection in remote sensing images. Netw. Secur. Data Gov. 41, 67–73. 10.19358/j.issn.2097-1788.2022.03.011

[B39] Zhang J. Wang F. Zhang H. Shi X. (2023). A novel CS 2G-starlet denoising method for high noise astronomical image. Optics Laser Technol. 163:109334. 10.1016/j.optlastec.2023.109334

